# Dropwort (Filipendula hexapetala Gilib.): potential role as antioxidant and antimicrobial agent

**DOI:** 10.17179/excli2014-479

**Published:** 2015-01-06

**Authors:** Jelena Katanic, Vladimir Mihailovic, Nevena Stankovic, Tatjana Boroja, Milan Mladenovic, Slavica Solujic, Milan S. Stankovic, Miroslav M. Vrvic

**Affiliations:** 1 Department of Chemistry, Faculty of Science, University of Kragujevac, Radoja Domanovica 12, 34000 Kragujevac, Serbia; 2 Department of Biology and Ecology, Faculty of Science, University of Kragujevac, Radoja Domanovica 12, 34000 Kragujevac, Serbia; 3 Faculty of Chemistry, University of Belgrade, Studentski trg 16, P.O. Box 51, 11158 Belgrade, Serbia

**Keywords:** Filipendula hexapetala Gilib., phenolic compounds, antioxidant activity, stability, in vitro digestion, antimicrobial activity

## Abstract

The aim of this study was to investigate the antioxidant activity of the methanolic extracts of *Filipendula hexapetala* Gilib. aerial parts (FHA) and roots (FHR) and their potential in different model systems, as well as antimicrobial activity. According to this, a number of assays were employed to evaluate the antioxidant and antimicrobial potential of *F. hexapetala* extracts. In addition, the antioxidant activity assays in different model systems were carried out, as well as pH, thermal and gastrointestinal stability studies. The phenolic compounds contents in FHA and FHR were also determined. The results showed that F. hexapetala extracts had considerable antioxidant activity *in vitro* and a great stability in different conditions. The extracts exhibited antimicrobial activity against most of the tested bacterial and fungal species. Also, the extracts contain high level of phenolic compounds, especially aerial parts extract.

## Introduction

Reactive oxygen species (ROS) produced as a result of an imbalance inside the cell are believed to be greatly responsible for the development of many diseases, like chronic inflammations, heart disease, neurodegenera¬tive disorders, and cancer (Lin and Chang, 2013[16]). In a biological system, the main components which prevent or markedly delay oxidation of substrate (carbohydrates, proteins, lipids and DNA) are commonly known as antioxidants. Their physiological role is to prevent damage of the cellular components (Niciforovic et al., 2010[22]). Food is a multi-component system composed of different biomolecules which could react with ROS. In this case, antioxidants could be used as food additives to preserve food by retarding deterioration, rancidity, or discoloration due to the oxidation (Wanasundara and Shahidi, 2005[40]). Since many herbs, plants, vegetables, and fruits extracts or powders have antioxidant properties, there is an increasing interest in natural sources of antioxidant molecules for use in the food, because the synthetic antioxidants, such as butylated hydroxyanisole (BHA) and butylated hydroxytoluene (BHT), accumulate in the body and could cause extremely harmful and destructive effects as potential carcino-gens (Niciforovic et al., 2010[22]; Mariem et al., 2014[18]). Antioxidants from natural sources could manifest their activity trough several mechanisms, like ability to scavenge free radicals, decompose them, to quench singlet oxygen, to act as a metal chelators or a synergists with the other components present (Nithiyanantham et al., 2013[23]).

In recent years, the most important problem that demands an appropriate solution is a resistance to antimicrobial agents. There is a wide range of microbial organisms that could not be treated with already known classes of drugs, because of this difficulty. The natural products, mainly plant extracts and essential oils, as rich sources of anti-infective agents, represent an alternative strategy for the treatment of the bacterial or fungal infections (Cushnie and Lamb, 2005[3]). The phenolic compounds in plants represent the main class of natural antioxidants and antimicrobial agents. The mechanisms responsible for the phenolic toxicity to microorganisms include: adsorption and disruption of microbial membranes, interaction with enzymes, and metal ion deprivation (Tajkarimi et al., 2010[36]).

*Filipendula hexapetala* Gilib. (dropwort, syn. *Filipendula vulgaris* Moench) belongs to genus *Filipendula* (Rosaceae), beside the meadowsweet (*Filipendula ulmaria* Maxim.) which is well known plant used in traditional medicine. Dropwort has a long history of use in folk medicine and phytotherapy in many European countries such as Serbia (Tucakov, 1973[37]), Poland (Oszmianski et al., 2007[25]), Russia (Olennikov and Kruglova, 2013[24]) and Romania (Imbrea et al., 2010[11]). The plant produces the rosette of fern-like, odd-pinnate leaves, and flowering shoots up to 80 cm high. The underground organs consist of rhizomes with tuberous roots. The crushed leaves and roots have a scent of oil of wintergreen (methyl salicylate) (Imbrea et al., 2010[11]). The entire plant or its individual parts are used in folk medicine. Powdered roots of *F. hexapetala* were utilized to treat kidney problems, breathlessness, wheezing, sore throats and congestion, stomachache and diarrhea (Tucakov, 1973[37]). The dropwort flowers have a long record of use in cases of rheumatism as a diuretic, or as an astringent in hemorrhoids, bleedings, etc. Nevertheless, many other properties including easing influenza and gout, and using a tea made from the leaves to cleanse wounds and sore eyes, i.e. anti-inflammatory, antipyretic, analgesic and antirheumatic properties have been reported (Tucakov, 1973[37]). Previously isolated classes of constituents in *F. hexapetala* are tannins (rugosins, tellimagrandins), phenolic acids (ellagic acid, gallic acid, caffeic acid, salicylic acid) and flavonoids (quercetin, hyperoside, kaempferol, apigenin, luteolin, dihydroquercetin, isoquercitrin, avicularin, spiraeoside, rutin) (Oszmianski et al., 2007[25]; Vengerovsky et al., 2011[39]; Olennikov and Kruglova, 2013[24]).

*F. hexapetala* contains high concentrations of phenolics, responsible for some of its antibacterial and antioxidant activity (Maksimovic et al., 2007[17]; Imbrea et al., 2010[11]). So far, there have been no detailed studies of the antioxidant and antimicrobial activity of extracts of aerial parts and roots, as well as their potential use as antioxidants in food products. The aim of this study was to examine *in vitro* antioxidant, antibacterial and antifungal activities of the methanolic extracts of *Filipendula hexapetala* Gilib. aerial parts (FHA) and roots (FHR), and their antioxidative potential against lipid peroxidation in different model systems, especially in the meat model system. The total contents of phenolic compounds were determined, as well as the stability of the extracts.

## Materials and Methods

### Chemicals

All chemicals and reagents were of analytical grade and were purchased from Sigma Chemical Co. (St. Louis, MQ, USA), Aldrich Chemical Co. (Steinheim, Germany) and Alfa Aesar (Karlsruhe, Germany). All spectrophotometric measurements were performed on UV–VIS double beam spectrophotometer Halo DB-20S (Dynamica GmbH, Switzerland).

### Plant collection and preparation of the methanol extracts

The roots and above-ground parts of *Filipendula hexapetala* Gilib. were collected at the locality Šumarice, (Kragujevac, Central Serbia), during the flowering season (May, 2013). Voucher specimen (No. 111/013) was prepared and deposited in the Herbarium of the Department of Biology and Ecology, Faculty of Science, University of Kragujevac, Kragujevac, Serbia, after the identification of species. The air-dried aerial parts (58 g) and roots (110 g) of *F. hexapetala* were fine powdered and separately extracted for 24 h with methanol for three times (500 ml each) at room temperature. After filtration through Whatman No. 1 filter paper, the extracts were concentrated in a rotary evaporator under reduced pressure. The percentage yields of methanolic extracts of the aerial parts (FHA) and roots (FHR) were 20.52 % (w/w) and 31.82 % (w/w), respectively. The concentrations used in the experiments were based on the dry weight of the extracts.

### Spectrophotometric determination of phenolic compounds content

The total phenolic content in plant extracts were estimated according to the Folin-Ciocalteu spectrophotometric method (Singleton et al., 1999[33]). Total phenols were determined as gallic acid equivalents (mg GAE/g extract).

The amounts of total flavonoids and flavonols were determined by spectrophotometric aluminum chloride methods according to Brighente et al. (2007[2]) and Yermakov et al. (1987[46]). The contents of flavonoids and flavonols were calculated as rutin equivalents (mg RUE/g dry extract).

Total phenolic (hydroxycinnamic) acids were determined by the Polish Pharmacopoeia method from the monograph of dandelion leaves (2005[27]). Briefly, 1 ml of extract (1 mg/ml) was added to 5 ml water, followed by 1 ml of 0.1 M HCl, 1 ml of Arnow reagent (10 % w/v of sodium molybdate and 10 % w/v sodium nitrite), 1 ml 1 M NaOH, filled up to 10 ml in a volumetric flask and the absorbance read immediately at 490 nm. The results were expressed as caffeic acid equivalents (mg CAE/g extract).

The method for determination of condensed tannins content relies on the precipitation of proanthocyanidins with formaldehyde (Scalbert et al., 1989[31]). First, the total phenolic content was measured as described before. A 0.5 mole equivalent of phloroglucinol was added for every gallic acid equivalent in the extract. To 2 ml of this plant extract and phloroglucinol was added 1 ml of a 2:5 HCl/H_2_O solution and 1 ml of an aqueous solution of formaldehyde (13 ml of 37 % formaldehyde diluted to 100 ml in water). After an overnight incubation at room temperature, the unprecipitated phenols were estimated in the supernatant by the Folin-Ciocalteu method. The precipitate contains the proanthocyanidins and known amount of phloroglucinol, which is always quantitatively precipitated. Content of condensed tannins were expressed as the difference between total phenolic content and unprecipitated phenols as gallic acid equivalents (mg GAE/g extract).

The content of gallotannins was determined by Haslam (1965[8]) method. The assay is based on the reaction of potassium iodate (KIO_3_) with galloyl esters, which will form a red intermediate and ultimately a yellow compound. Gallotannins content was determined as gallic acid equivalents (mg GAE/g extract).

The spectrophotometric pH differential method (Giusti and Wrolstad, 2003[6]) was used to determine monomeric and total (monomeric plus polymerized) anthocyanins. Two dilutions of the same sample were prepared in 0.025 M potassium chloride solution and in 0.4 M sodium acetate solution adjusted respectively to pH 1.0 and 4.5 with HCl. The absorbance of each dilution was measured at 520 and 700 nm against distilled water blank. Absorbance measurements were taken at the wavelength of maximum absorbance of the pH 1.0 solution. The difference in absorbance between the two buffer solutions is due to the monomeric anthocyanin pigments. Monomeric and total anthocyanin contents were expressed as cyanidin-3-glucoside equivalents (mg cyanidin-3-glucoside/g dry extract). Absorbance (A) was calculated as follows:

A = (A_λmax - A700_) _pH=1.0_ - (A_λmax - A700_) _pH=4.5_; where λ_max_ = 520 nm.

The monomeric anthocyanin concentration in the original sample was obtained from the following equation (1):

[monomeric anthocyanins] (mg/l)=(A×MW×DF×1000)/(ε×1) (1)

The total anthocyanin concentration was obtained from the equation (2) below: 

[total anthocyanins] (mg/l)=(A'×MW×DF×1000)/(ε×1) (2)

where:

A' = (A_λmax_ - A_700_) _pH=1.0_; MW =molecular weight (449.2 g/mol cyanidin-3-glucoside); DF =dilution factor; ε = molar extinction coefficient, l x mol^–1^ x cm^–1^ (26900 l mol^-1 ^cm^-1 ^cyanidin-3-glucoside); l = path length (1 cm).

All determinations of phenolic compounds were carried out in triplicates.

### In vitro antioxidant activity

#### Determination of total antioxidant capacity

The total antioxidant activity of the methanol extracts was evaluated by the phosphomolybdenum method (Prieto et al. 1999[28]). The assay is based on the reduction of Mo(VI) - Mo(V) by the antioxidant compounds and formation of a green phosphate/Mo (V) complex at acid pH. 0.3 ml of extracts samples were added to 3 ml of reagent solution (0.6 M sulfuric acid, 28 mM sodium phosphate and 4 mM ammonium molybdate). The tubes were incubated at 95 °C for 90 min and after cooling to room temperature the absorbance was measured at 695 nm. The total antioxidant capacity was expressed as milligrams of ascorbic acid (AA) per gram of the dry extract.

#### Determination of ABTS radical-cation scavenging activity

The antioxidant capacity was estimated in terms of the ABTS·^+ ^radical-cation scavenging activity following the procedure described by Re et al. (1999[29]). ABTS·^+^ was obtained by reacting 7 mM ABTS stock solution with 2.45 mM potassium persulfate and the mixture was left to stand in the dark at room temperature for 16 h before use. The ABTS·^+^ solution (stable for 2 days) was diluted to an absorbance at 734 nm of 0.70 ± 0.02. 100 µl of extract was added to 900 µl of diluted ABTS·^+ ^solution and the absorbance was measured after 30 min. Gallic acid (GA), ascorbic acid (AA), rutin (RU) and butylated hydroxytoluene (BHT) were used as reference antioxidants. All samples were analyzed in triplicate. The ABTS·^+ ^radical-cation scavenging activity of the samples was expressed as: 

% radical scavenging activity = [(A_C_ – A_S_) / A_C_] x 100,

where A_C_ is the absorbance of the blank control (ABTS·^+ ^solution without test sample) and A_S_ is the absorbance of the test sample.

#### Determination of DPPH free-radical scavenging activity

The used method by Kumarasamy et al. (2007[14]) was adopted with suitable modifications. Serial dilutions of the extracts (2 ml each, in methanol) were mixed with DPPH (2 ml, 80 µg/ml, in methanol) and the absorbance was measured after 30 min at 517 nm. Ascorbic acid, GA, RU and BHT were used as reference standards. The DPPH free-radical scavenging activity (%) was calculated using the following equation: 

% radical scavenging activity = [(A_C_ – A_S_) / A_C_] x 100.

The IC_50_ value, which is the concentration of the test material that reduces 50 % of the free-radical concentration, was calculated as µg/ml through sigmoidal dose-response curve.

#### Measurement of reducing power

The reducing power was determined according to the method of Oyaizu (1986[26]). Various concentrations of methanolic extracts (2.5 ml) were mixed with sodium phosphate buffer (2.5 ml, 200 mM, pH 6.6) and potassium ferricyanide (2.5 ml, 1 %). The mixtures were incubated at 50 °C for 20 min. After trichloroacetic acid were added (2.5 ml, 10 %), the mixtures were centrifuged at 1000 rpm for 8 min. The upper layer (5 ml) was mixed with ferric chloride (1 ml, 0.1 %) and the absorbance was measured spectrophotometrically at 700 nm. GA, AA, RU and BHT were used as standards. A higher absorbance of this mixture indicates a higher reducing activity.

#### Measurement of ferrous ion chelating ability

The ferrous ion chelating activity of the extracts was measured by the decrease in absorbance at 562 nm of the iron (II)-ferrozine complex (Yan et al., 2006[43]). 1 ml of 0.125 mM FeSO_4_ was added to 1.0 ml sample (with different dilutions), followed by ferrozine (1.0 ml, 0.3125 mM). After 10 min the absorbances of the mixtures were measured. GA, RU and ethylenediaminetetraacetic acid (EDTA) were used as standards. The ability of the sample to chelate ferrous ion was calculated relative to the control (consisting of iron and ferrozine only) using the formula:

% chelating effect = [(A_C_ – A_S_) / A_C_] x 100.

#### Superoxide radical scavenging activity by alkaline DMSO method

Superoxide radical scavenging activity of the extracts was determined by the alkaline DMSO method according to Kunchandy and Rao (1990[15]) with slight modifications. Superoxide radicals were generated by the addition of sodium hydroxide to air saturated dimethyl sulfoxide (DMSO). The generated superoxide remains stable in solution and reduces nitroblue tetrazolium (NBT) into formazan dye at room temperature which can be measured at 560 nm. GA, AA, RU and BHT were used as standards.

### Antioxidant activity in different model systems

#### Determination of the inhibitory activity toward lipid peroxidation (oil-in-water emulsion)

The thiocyanate method (Hsu et al., 2008[9]) was used to determine antioxidant activity of extracts in oil-in-water emulsion. 0.5 ml of each extract and standard solution was added to 2.5 ml of linoleic acid emulsion (0.2804 g linoleic acid, 0.2804 g Tween-80 as emulsifier in 50 ml 40 mM phosphate buffer, pH 7.0) and then the mixture was homogenized. The final volume was adjusted to 5 ml with 40 mM phosphate buffer, pH 7.0. After incubation at 37 °C in the dark for 72 h, a 0.1 ml aliquot of the reaction solution was mixed with methanol (4.7 ml, 75 %), FeSO_4_ (0.1 ml, 20 mM) and ammonium thiocyanate (0.1 ml, 30 %). The absorbance of this mixture was measured at 500 nm, after 3 min of stirring. GA, AA, RU, α-tocopherol and BHT were used as the reference compounds. Inhibition percent of linoleic acid peroxidation was calculated using the following formula:

% inhibition = [(A_C_ – A_S_) / A_C_] x 100.

#### ß-Carotene-linoleic acid model system

The lipid antioxidant activities of the samples were also determined using ß-carotene-linoleic acid bleaching assay (Wu et al., 2010[42]). A solution of ß-carotene was prepared by dissolving ß-carotene (2 mg) in chloroform (10 ml). The ß-carotene-chloroform solution (2 ml) was pipetted into a round-bottomed flask and chloroform was removed using a rotary evaporator at 40 °C for 5 min. Thereafter, 40 mg of linoleic acid, 400 mg of Tween 40 emulsifier, and 100 ml of distilled water were added to the flask with vigorous agitation to form an emulsion. The aliquots (4.8 ml) of this emulsion were added into test tubes containing different concentrations of sample solutions (0.2 ml), and the absorbance was immediately measured at 470 nm against a blank, consisting of an emulsion without ß-carotene. The tubes were incubated in a water bath at 50 °C, and the absorbance of oxidative emulsion was measured at 470 nm over a 60 min period by a spectrophotometer. Control samples contained 0.2 ml of water instead. GA, RU and a-tocopherol were used as the reference compounds. The antioxidant activity was expressed as an inhibition percentage with reference to the control after a 60 min incubation using the following equation: 

AA = [(DRC – DRS) / DRC] x 100,

where AA = antioxidant activity, DRC = degradation rate of the control = [ln (a/b) / 60], DRS = degradation rate in presence of the sample = [ln (a/b) / 60], a = absorbance at time 0, and b = absorbance at 60 min.

#### Meat model system

The inhibition of lipid peroxidation in the meat model system was determined according to Wettasinghe and Shahidi (1999[41]) with a slight modification. Fresh pork was acquired from a local supermarket and most of its surface fat was removed. The meat was ground twice in a meat grinder, divided into six equal parts (300 g each) and every part was mixed with 20 % by weight of deionized water. Plant extracts (100 and 500 mg/kg) were added directly to the meat. BHT as a standard synthetic antioxidant (in a concentration of 50 mg/kg) was added to the one part of the meat. A control sample containing no extract was also prepared. Meat systems were homogenized, transferred into plastic pans and then stored for 14 days at 4 °C. After designated time (0, 1, 2, 3, 7, 9, 11 and 14 days), the samples were randomly taken for TBARS determination according to the Siu and Draper (1978[34]) method. Meat samples (2 g) were mixed with trichloroacetic acid (TCA, 5 ml, 10 %) and vortexed for 2 min. TBA reagent (5 ml) was added to the tube and vortexed for 30 s. Hereupon, the samples were centrifuged at 4000 rpm for 10 min, the supernatants were removed in the tubes which then were placed in a boiling water bath for 45 min, cooled to room temperature in ice, and the absorbance value of TBA-malonaldehyde adduct was read at 532 nm. The TBARS values were then calculated using the standard curve of malondialdehyde (MDA) and expressed as mg MDA equivalents/kg sample.

### Stability studies of the plant extracts

#### pH stability 

The pH stability study was determined according to the Kittiphattanabawon et al. (2012[13]). The plant extracts were dissolved in distilled water (25 ml), previously adjusted to the different pH values (1, 3, 5, 7 and 9) using 1 M HCl or 1 M NaOH, to obtain a final concentration of 2 mg/ml. The mixtures were incubated at the room temperature for 1 h. Thereafter, the pH of the mixtures was adjusted to 7.0 and their volumes were made up to 50 ml with distilled water. The residual antioxidant activity was determined using the measurement of total phenolic content (TPC) and DPPH scavenger assay expressed as the activity (%) relative to that obtained without pH adjustment.

#### Thermal stability

Thermal stability of the extracts was estimated following the procedure described by Kittiphattanabawon et al. (2012[13]). The plant extract was dissolved in 50 ml distilled water (pH 7) to obtain a final concentration of 1 mg/ml. 10 ml of the solution was transferred to a screw-capped test tube which were placed in a boiling water bath (100 °C) for 0, 15, 30, 60, 120, 180 and 240 min. After designated heating times, treated samples were immediately cooled in the iced water. The residual antioxidant activity was determined using the measurement of total phenolic content (TPC) and DPPH scavenger assay expressed as the activity (%) relative to those without heat treatment.

#### Stability in gastrointestinal tract model system

To simulate the *in vivo* resistance to digestion method described by Enari et al. (2008[4]) with slight modifications was used. The extracts solutions (1 mg/ml; 100 ml) were mixed with 10 ml of 10 mM phosphate buffer (pH 6.8). The mixtures were incubated for 2 min at 37 °C (oral condition). Then 0.5 ml of 1 M HCl–KCl buffer (pH 1.5) was added to produce an acidic condition (pH 1.5), followed by adding 32 U/ ml of pepsin solution in 1 M HCl–KCl buffer (pH 1.5) (5 ml) and incubating for 60 min at 37 °C (stomach condition). Thereafter, the pH was adjusted to 6.8 with 1 M NaHCO3 (1 ml), and the enzyme mixture of bile and pancreatic juice (1 ml) that contained pancreatin (10 mg/ml), trypsin (14 600 U/ml) and bile extract (13.5 mg/ml) in 10 mM phosphate buffer (pH 8.2), was added to the solution, followed by incubation at 37 °C for 3 h (duodenal condition). To investigate the changes in total phenolic content (TPC) and DPPH scavenger activity of the FHA and FHR extracts during the simulated gastrointestinal digestion, aliquots of gastrointestinal digests were randomly taken at 0, 0.5, 1, 2, 3 and 4 h during the *in vitro *digestion. To inactivate duodenal enzymes, the test tubes were immediately placed in the boiling water for 10 min. The residual antioxidant activity was determined using the measurement of TPC and DPPH scavenger assay and expressed as the activity (%) relative to those without any treatment.

### Antimicrobial activity

#### Microbial strains

The antibacterial activity of extracts of *F. hexapetala* aerial parts (FHA) and roots (FHR) was individually tested against a panel of three ATCC bacterial strains and three clinical isolates. For the antifungal activity assay were used 10 isolates and *C. albicans* (ATCC 10259). Following microbial strains were used in this investigation: bacterial strains *Klebsiella pneumoniae* (ATCC 70063), *Pseudomonas aeruginosa* (ATCC 10145), *Escherichia coli* (ATCC 25922), *Escherichia coli* (isolate), *Enterococcus faecalis* (FSB 24) and *Pseudomonas aeruginosa* (FSB 37); test fungi were *C. albicans* (ATCC 10259), *Trichoderma harzianum* (FSB 12), *Trichoderma longibrachiatum* (FSB 13), *Penicillium cyclopium *(FSB 23), *Penicillium canescens* (FSB 24), *Aspergillus niger* (FSB 31), *Aspergillus glaucus* (FSB 32), *Fusarium oxysporum* (FSB 91), *Alternaria alternata* (FSB 51), *Doratomyces stemonitis* (FSB 41) and *Phialophora fastigiata* (FSB 81).

All test organisms were obtained from the Faculty of Biochemistry and Chemistry, University of Belgrade and Laboratory for Microbiology, Department of Biology, Faculty of Science, University of Kragujevac, Kragujevac, Serbia. Bacteria were maintained on nutritient agar and fungi cultures were maintained on potato-dextrose agar excluding *C. albicans* which was maintained on Sabouraund dextrose agar. The cultures were stored at +4 °C and subcultured once a month.

#### Determination of minimum inhibitory concentration (MIC)

The minimum inhibitory concentrations (MICs) of *F. hexapetala* extracts (FHA and FHR) against tested microorganisms were determined according to the microdilution method (Sarker et al., 2007[30]). For all the tests with bacterial strains was used the Mueller–Hinton broth (MHB) and for determination of antifungal activity the Sabouraud dextrose broth (SDB) was used. The assay for MICs determining was performed by a serial dilution technique using 96-well microtiter plates. The investigated extracts (10.00-0.078 mg/ml) and phenolic compounds gallic acid and quercetin (1.00-0.0078 mg/ml) were added into the first row of the plate and in all the other rows that were filled with 50 µl of MHB or SDB, and the double dilutions were made. Thereafter, 10 µl of resazurin indicator solution (270 mg resazurin in 40 ml of sterile distilled water) and 30 µl of nutrient broth were added to the each well. A 10 µl of SDB was added in tests with fungi instead of resazurin solution. Finally, 10 µl of bacterial suspension (1.0 × 10^6^ CFU/ml) or yeast spore suspension (1.0 × 10^4^ CFU/ml) was added to the each well. For each strain, the growth conditions and the sterility of the medium were checked. The microplates were incubated for 24 h at 37 °C for the bacteria, or for 48 h at 28 °C for fungi, respectively. Color change was then assessed visually and any color change from purple to pink or colorless was recorded as positive. The lowest concentration with no observed color change was taken as the MICs value for bacterial strains and the lowest concentrations without visible growth were defined as MICs for fungi. All tests were repeated in triplicate. Amracin (tetracycline) (10-0.078 µg/ml) was used as a referent antibiotic, while nystatin and fluconazole (10-0.078 µg/ml) were used as a positive control for antifungal activity.

### Statistical analysis

The data are expressed as mean ± standard deviation (SD). The IC_50_ for *in vitro* antioxidant potential was calculated using nonlinear regression analysis from the sigmoidal dose-response inhibition curve. Statistical analyses of the data were analyzed using analysis of variance (ANOVA) and the group means were compared by the least significant difference test (LSD). The results were considered statistically signiifcant if the *P* < 0.05.

## Results and Discussion

### Phytochemical results

The phenolic compounds from plant extracts often display antioxidant activity and their content can be used as an important indicator of antioxidant capacity of various plant species. The results of the phenolic compounds content determination in the selected plant extracts are presented in Table 1(Tab. 1).

The content of total phenolics in FHA, evaluated by Folin-Ciocalteu method and expressed as gallic acid equivalents (GAE), was higher (267.20 mg GAE/g dry extract) than in FHR (219.20 mg GAE/g dry extract). The results of total flavonoid and flavonols contents determination (expressed as rutin equivalents: mg RU/g of dry extract), presented in Table 1(Tab. 1), showed multiple higher level of flavonoid and flavonols contents in FHA than in FHR. Contrary, much higher level of total phenolic acids (hydroxycinnamic acids) was found in root extract than in aerial parts extract. The hydroxycinnamic acids levels in FHA and FHR were 31.29 and 55.52 mg CAE/g, respectively. The results showed that the most abundant phenolic compounds in root extract are condensed tannins and gallotannins (213.71 and 48.53 mg GAE/g dry extract, respectively), while the aerial parts extract contained lower amounts (87.55 and 33.67 mg GAE/g dry extract, respectively). The monomeric and total anthocyanin contents in the extracts (Table 1(Tab. 1)) expressed as cyanidin-3-glucoside equivalents (mg Cy 3-glc/g dry extract), were negligible low, although with slightly higher values in FHA (3.34 and 4.50 mg Cy 3-glc/g, respectively). According to the results obtained in the investigation of phenolic compound contents, it is obvious that the root extract contained tannins in high percentage, while aerial parts extract contained higher concentration of total flavonoids compared to the other groups of phenolic compounds. The findings of our study are in consistent with previous reports that leaves and flowers of *F. hexapetala* contains high amount of flavonoids (Olennikov and Kruglova, 2013[24]), while roots contain high level of tannins (Oszmianski et al., 2007[25]).

### In vitro antioxidant activity

For determining antioxidant activity of plant extracts are in use a huge variety of methods, but each one has its own limitations due to the complex nature of phytochemicals and their interactions. Therefore, the importance of using multi-assay systems is well argued (Mihailovic et al., 2013[19]). For a more comprehensive and accurate evaluation of the antioxidant potential of *F. hexapetala* extracts, a range of six different *in vitro* assays were carried out. The results for antioxidant activity of tested plant extracts and standard natural and synthetic compounds are presented in Table 2(Tab. 2).

The phosphomolybdenum method, an assay based on the reduction of Mo (VI) - Mo (V) by the antioxidant compounds, is quantitative since the antioxidant activity is expressed as the number of equivalents of ascorbic acid (AA) per gram of dry extract (Prieto et al. 1999[28]). FHA and FHR extracts showed a high total antioxidant capacity, with 434.89 and 495.33 mg AA/g dry extract, respectively (Table 2(Tab. 2)).

The ABTS^+•^ and DPPH radical have been widely used to test the free radical scavenging ability and to evaluate the antioxidative activity of plant extracts and foods. The results of these two assays are presented in Table 2(Tab. 2) and lower IC_50_ values reflects better ABTS^+•^ and DPPH radical scavenging activities. In the assay with ABTS^+•^, IC_50_ values of FHA and FHR were not significantly different (*P* > 0.05) and compared to rutin, as a natural antioxidant compound, the extracts showed better antioxidant properties (*P* < 0.05). The ABTS radical scavenging activity decreased in the order of gallic acid > BHT > ascorbic acid > FHA > FHR > rutin. At the concentration of 13.47 µg/ml, FHA was able to inhibit 50 % of the DPPH^•^ radical, which was 2-fold less concentration than FHR (26.27 µg/ml) and not significantly different (*P* > 0.05) in regard to synthetic antioxidant compound BHT (Table 2(Tab. 2)). FHA extract showed significantly (*P* < 0.05) better DPPH^•^ radical scavenging activity than rutin, which IC_50_ value was 23.13 µg/ml. The DPPH^•^ scavenging activity of extracts compared to natural and synthetic antioxidant compounds, in the order of activity, was as follows: gallic acid > ascorbic acid > FHA > BHT > rutin > FHR (Table 2(Tab. 2)), and the rank order was similar with ABTS^+•^ assay. In the ABTS^+•^ and DPPH assays, FHA extract showed better antioxidant activities than rutin. Therefore, it revealed that the aerial part extract was a high potential natural antioxidant. Ozmianski et al. (2007[25]) reported that the roots of *F. hexapetala* possess very good ABTS^+•^ scavenger activity (1.86 µM trolox/100 g dried roots) compared to the some other Rosaceae plant roots. The DPPH scavenging activity values agree rather well with other published data. In a study carried out with *F. hexapet*ala flower extract, Maksimovic et al. (2007[17]) found that it had very good DPPH radical scavenging activity compared to the quercetin.

**Figure 1:**
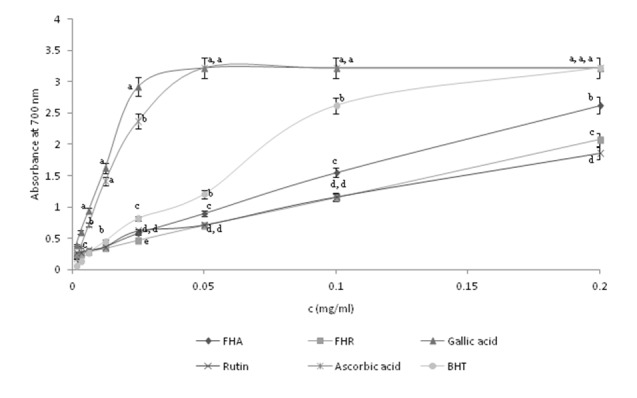
Figure 1: Reducing power of selected plant extracts compared to reducing power of standards at different concentrations. Each value is the average of three measurements with error bars representing SD. Means followed by different letters are significantly different at *P*<0.05, for the same concentrations of extracts and standards.

The ferric reducing antioxidant power assay, characterized by the reduction of Fe^3+^ to Fe^2+^, is frequently employed to assess the efficiency of antioxidants for their electron transfer capacity and could, therefore, serve as a significant indicator of its antioxidant activity (Yan et al., 2006[43]). An increase in absorbance of the reaction mixture which color changes from yellow to blue indicates an increase in the reducing capacity due to an increase in the formation of the complex. Figure 1(Fig. 1) shows the reductive capabilities of aerial parts and roots methanol extracts of *F. hexapetala*, compared to gallic acid, ascorbic acid, rutin and BHT. It can be noticed on Figure 1(Fig. 1) that the FHA and FHR extracts possess certain reducing capacity, but they were less effective compared to standard antioxidant compounds. FHA possess better reducing power, in all applied concentrations, compared to FHR and rutin. At the concentrations of 0.1 and 0.05 µg/ml, FHR and rutin showed almost identical reducing activity.

Ferrous ions are the most effective pro-oxidants and they are commonly found in food systems, where they can initiate lipid peroxidation and start a chain reaction that leads to the deterioration of food. Their interaction with hydrogen peroxide in biological systems can lead to formation of greatly reactive hydroxyl radicals. These processes can be delayed by chelation of iron ion. Particularly, the phenolic compounds bear hydroxyl and carboxyl groups, able to bind iron and copper (Yanishlieva-Maslarova, 2001[44]). The examined extracts, as well as antioxidant standards used (GA and RU) showed weak ability to chelate ferrous ions compared to EDTA (IC_50_ = 2.69 µg/ml).

Superoxide anion (O_2_^•-^) is the most common free radical *in vivo*, a precursor for other ROS that have the potential reactivity with biological molecules. Therefore, the reduction of superoxide anions concentration is very important for organism under conditions of oxidative stress. In applied NBT assay superoxide radical scavenging activity of the extracts was observed in comparison with standard antioxidants (GA, AA, RU and BHT) and results are presented in Table 2(Tab. 2). The O_2_^•-^ scavenging action of FHR was considerable (IC_50_ = 432.27 µg/ml) compared to the well-known antioxidant ascorbic acid (IC_50_ = 778.89 µg/ml) and BHT (IC_50_ > 1000 µg/ml). FHA and all standards except GA showed lower O_2_^•- ^ scavenging activity than FHR. Considering that both extracts, especially FHR, showed high level of neutralization of this biologically important radical compared to the pure antioxidant compounds, it revealed that extracts were highly potent natural antioxidants.

According to the results, *F. hexapetala* extracts can effectively scavenge different types of free radicals or reactive oxygen species under *in vitro* conditions (Table 2(Tab. 2)). The broad range of results indicates that multiple mechanisms may be responsible for their antioxidant capacity, related to their phenolic composition (Yao et al., 2013[45]). Although all the antioxidant methods have a different nature and origin among them, *F. hexapetala* extracts followed a similar trend in ABTS, DPPH and ferric reducing antioxidant assays, suggesting that FHA extract is more abundant in antioxidants. Also, it should be noted that, although we observed that the extract with higher level of total phenolics, mainly flavonoids and flavonols, had better antioxidant properties, *F. hexapetala* extracts might contain other compounds that could contribute to their overall antioxidant potential.

### Antioxidant activity in different model systems

#### Inhibitory activity toward lipid peroxidation (oil-in-water emulsion)

Besides the role of natural antioxidants as protective compounds against disease, they also have an important role in the prevention of oxidative deterioration of lipid food. Oxidation of lipids in food products affects the color, flavor, texture, nutritive value of foods and may cause damaging conditions following consumption of potentially toxic reaction products (Wettashinge and Shahidi, 1999[41]). Lipid peroxidation involves a chain reaction mechanism which is autocatalytic in nature, but it can be inhibited by using standard antioxidants, compounds isolated from the plants, or plant extracts which possess antioxidant activity. It can be used a number of methods to evaluate potential inhibitory activity of extracts toward lipid peroxidation (Mariem et al., 2014[18]; Gonçalves et al., 2013[7]).

Polyunsaturated fatty acids, such as linoleic acid, are easily oxidized by oxygen in the air. This auto-oxidation leads to the formation of linoleic acid peroxides. Tested extracts added to the linoleic acid emulsion were able to reduce the formation of hydroperoxide. The IC_50_ values of the FHA and FHR extracts were 20.80 and 18.77 µg/ml, respectively. In the linoleic acid system were no significant difference (*P* > 0.05) in IC_50_ values between extracts, while the referent compounds α-tocopherol (IC_50_ = 0.51 µg/ml) and BHT (IC_50_ = 1 µg/ml) exhibited significantly (*P* < 0.05) lower IC_50_ values (Table 2(Tab. 2)). In comparison, both extracts showed much higher activity than referent compounds gallic acid and ascorbic acid. The highest inhibitory activity toward lipid peroxidation among all the tested samples was observed for the nonpolar BHT. These results together confirm the well-known phenomenon that the polar antioxidants remaining in the aqueous phase of the emulsion and thus are less effective in protecting the emulsified linoleic acid, whereas lipophilic anti-oxidants due to higher partition into the lipid phase reveal greater activity in the emulsion (Moure et al., 2001[20]).

#### ß-Carotene-linoleic acid bleaching assay

The ß-carotene-linoleic acid bleaching inhibition effect of *F. hexapetala* extract and referent compounds are shown in Table 2(Tab. 2). According to the results, FHA reduced the oxidation of ß-carotene much better (IC_50_ =93.96 µg/ml) than FHR extract (IC_50_ =263.83 µg/ml). ß-Carotene in this model system undergoes rapid discoloration in the absence of an antioxidant. This is because of the coupled oxidation of ß-carotene and linoleic acid, which generates free radicals. Neither of referent compounds inhibited the oxidation of linoleic acid, at tested concentrations. Both extracts were better in their effect on reducing the oxidation of ß-carotene than GA, RU and α-tocoferol.

**Figure 2:**
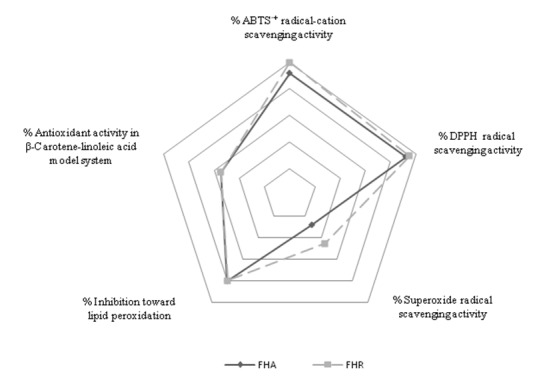
Comparison of radical scavenging activities and inhibition of lipid peroxidation of *F. hexapetala* extracts (FHA and FHR), at the concentration of 0.25 mg/ml, expressed in percent of inhibition.

The relationship between different radical scavenging activities of the dropwort extracts (at the concentration of 0.25 mg/ml) and effect toward lipid peroxidation is presented by radar diagram (Figure 2(Fig. 2)). It is obvious that both extracts possess almost equal percent of lipid peroxidation inhibition by previously described methods. However, much better results were recorded with radical scavenging methods (precisely ABTS and DPPH mehods), where % of scavenging activities of tested extracts were approximately 100 %, at the concentration of 0.25 mg dry extract/ml.

#### Meat model system

**Figure 3:**
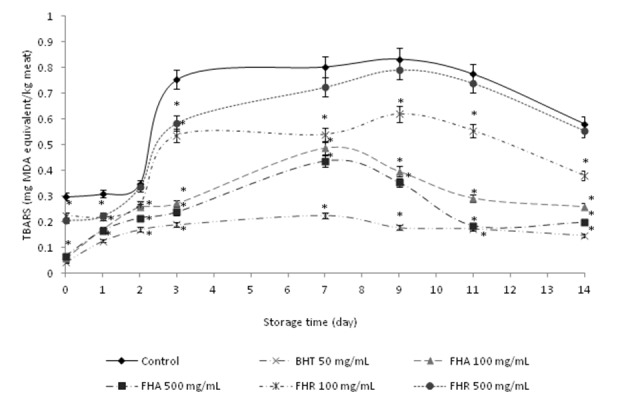
Changes in lipid oxidation of meat model system added with methanolic extracts of *F. hexapetala* aerial parts and roots (FHA and FHR) at different concentrations. Butylated hydroxytoluene (BHT) was used as referent synthetic antioxidant. Bars represent standard deviation (n = 3). *P<0.05 when compared with the control group (without an antioxidant).

In this study, fresh pork meat was used as a food model to determine whether the incorporation of *F. hexapetala* extracts (FHA and FHR), with high antioxidant activity, could effectively inhibit the meat lipid peroxidation during storage. Lipid oxidation is a prominent problem in food high in fat, and during storage secondary products formed by oxidation may react with biomolecules and exert cytotoxic and genotoxic effects. Among these products there is malondialdehyde (MDA), which is the most used as a marker of oxidative stress and lipid peroxidation index (Nasri et al., 2013[21]). MDA reacts with thiobarbituric acid (TBA) to give the TBA reactive substances (TBARS) detectable spectrophotometricaly at 532 nm. The levels of TBARS were analyzed in fresh pork meat prepared with or without antioxidant in different concentrations, in terms of determination the ability of tested plant ex-tracts to inhibit lipid peroxidation during food storage. As presented on Figure 3(Fig. 3), the results showed that TBARS values of the control increased as a function of storage time. In meat model system, FHA and FHR extracts exerted a concentration-dependent antioxidant effect. Both concentrations of the extracts resulted in lower TBARS values throughout the entire storage period, compared to the control sample without antioxidants. BHT at a concentration of 50 mg/kg exerted the greatest inhibition of TBARS formation, as observed on Figure 3(Fig. 3). Generally, the TBARS values of meat with and without antioxidants increased up to 7 days (for FHR and control group 9 days) of storage (*P* < 0.05). After 7 days (9 days) of storage at 4 °C, gradual decreases in TBARS values were observed until the end of storage (*P *< 0.05). This was probably due to the loss of oxidation products formed, particularly low molecular weight volatile compounds. MDA and other short-chain products of lipid oxidation are not stable for a long period of storage. Oxidation of these products yields alcohols and acids, which are not determined by the TBA test (Wettasinghe and Shahidi, 1999[41]; Nasri et al., 2013[21]). Interestingly, both concentrations of FHA and FHR assayed inhibited the TBARS formation during storage. On storage day 3, both concentrations of FHA reduced the meat lipid oxidation by more than 60 % as compared to the control (Figure 3(Fig. 3)). Meanwhile, the FHR extract also showed lower TBARS values compared to the control group, but less effective than FHA and BHT. The meat system containing higher levels of plant extracts had lower TBARS during the storage period (*P* < 0.05). In meat systems containing plant extracts the inhibition of TBARS production was most probably due to their antioxidant and radical scavenging activities. However, the lowest TBARS values during the storage time period of BHT (50 mg/kg) treated system showed that it had a better preventive effect on lipid peroxidation than examined plant extracts. The obtained results correspond to the study of Kim et al. (2013[12]), which results showed that adding phenolic-rich extracts protects beef patties against lipid oxidation and could be used as multifunctional preservatives in meat products.

### Stability studies of plant extracts

**Figure 4:**
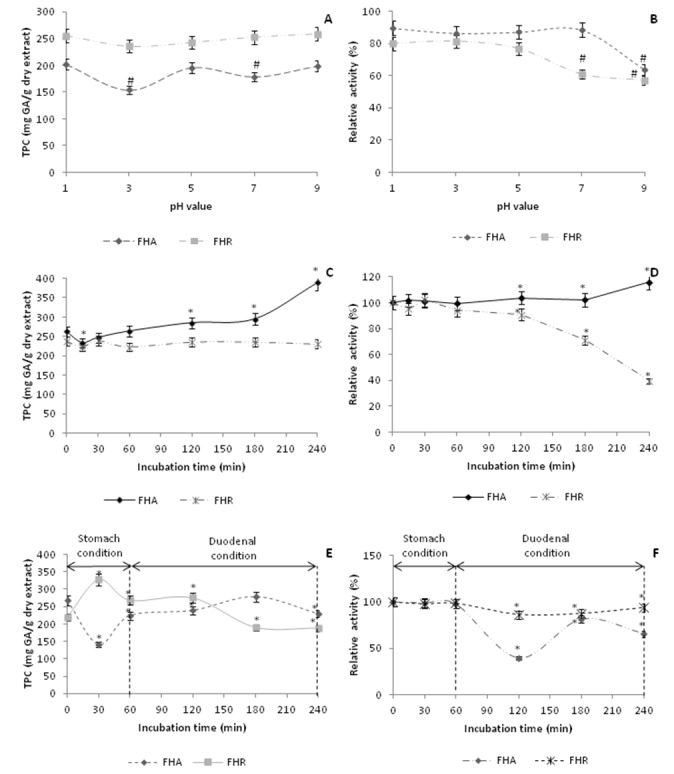
pH (A, B), thermal (C, D) and *in vitro* digestive (E, F) stabilities of methanolic extracts of *F. hexapetala* aerial parts and roots (FHA and FHR) with as monitored by the total phenolic content (TPC) and DPPH scavenger activity assays. Bars represent standard deviation (n = 3). # *P*<0.05 when compared with the untreated extracts. *P<0.05 when compared to the zero time (without any treatment).

Processing of plant material (medicinal plant, spices, fruits or vegetables) leads to changes in the contents of bioactive compounds and antioxidant activity (Im et al., 2011[10]). It was of interest to know the degree of such changes. Therefore, in this investigation were determined not only the contents of phenolic compounds and antioxidant activity of *F. hexapetala* extracts, but also these indices in the samples subjected to different pHs, boiling for 240 min at 100 °C and *in vitro* digestion. Simultaneously, the stability of the bioactive compounds and their antioxidant activity were studied. Figure 4(Fig. 4) shows the comparative diagrams of pH, thermal and digestive stabilities of tested dropwort extracts in terms of content of total phenolic compounds and relative antioxidant activity. It is of great importance to observe various types of stability in order to predict activity of the extracts in different conditions.

#### pH stability

The effect of pH on total phenolic content (TPC) and DPPH scavenging activity of tested plant extracts is shown in Figures 4A and 4B(Fig. 4). The TPC values of FHR were not significantly changed at all tested pH values. Meanwhile, TPC values of FHA were significantly changed at pH 3 and 7 (*P* < 0.05). The results of DPPH scavenging activity of FHA reported in Figure 4B(Fig. 4) indicated clearly that FHA extract was quite stable over the pH range of 1–7 (*P* > 0.05). At pH 9, the DPPH scavenging activity decreased compared with that found in the sample without pH adjustment. On the other hand, the DPPH scavenging activity of FHR showed better stability at pH range of 1–5. At pH 7–9 relative activity of FHR decreased by 40 % compared with sample without pH adjustment (100 %). Thus, it suggests that FHA and FHR had a potential for application in any food system over the pH range of 1–9 without any significant loss in activity. The acidic environment may induce hydrolysis and/or transformation of structure of the phenolic compounds. Literature data showed poor stability of phenolic acids to pH changes, while flavonoids were not affected by acid/alkali conditions (Friedman and Jürgens, 2000[5]). Since the FHA extracts was rich in flavonoids, this can be the reason of better relative antioxidant activity of this extract compared to FHR at different pHs.

#### Thermal stability

The processing stability of plant extracts after thermal treatment is required for their eventual incorporation in food formulations. Therefore, FHA and FHR extracts were subjected for 240 min at 100 °C and then the total phenolic content (TPC) and DPPH anti-oxidant activity were determined. The results are shown in Figure 4C and 4D(Fig. 4). No significant changes in the DPPH scavenging activity of both extracts were observed in the first 60 min of heating. Up to 240 min of heating it was noted that the DPPH scavenging activity of FHA increased and the activity of FHR decreased up to the end of heating time. In regard of total phenolic content, it was noticed that the TPC of both extract slightly decreased when heating time increased up to 15 min. Thereafter, the TPC of FHA increased up to 240 min of heating and TPC values of FHR were not significantly changed until the end of heating time. These results indicate that the examined extracts could be incorporated in any food subjected to thermal processed at 100 °C (up to 240 min for FHA and up to 120 min for FHR) with any loss of antioxidant activity, and with no significant change in total phenolic content values.

The change of TPC values in the thermal stability study follows the change of antioxidant activity. Greater stability of the FHA extract can be explained by the fact that the extract of aboveground parts contains a significant amount of flavonoids which are the most thermostable among all phenolic biocompounds. It has been reported in the literature that flavonoids, especially the flavan-3-ols, are more thermostable and the extra-stability in aqueous solution is due to intermolecular H-bonding (Im et al., 2011[10]).

#### Stability in gastrointestinal tract model system

The stability of the tested extracts, with high antioxidant activity, in the presence of digestive enzymes and duodenal juice was studied in order to predict their biological effects *in vivo*. The remaining antioxidant activities of the extracts, as monitored by TPC content and DPPH scavenging activity, after different digestion times, are presented in Figure 4E and 4F(Fig. 4). The DPPH activity of FHA and FHR were not significantly changed after pepsin digestion (until 60 min). Meanwhile, the TPC content value of FHA increased after pepsin digestion and the same value for FHR was significantly lower. Under duodenal condition, DPPH relative activities for FHR slightly decrease and remained constant to the end of digestion time. TPC values for FHR decreased to the end of digestion (time = 240 min). DPPH relative activities of FHA extract under duodenal conditions decreased to 120 min and then increased slightly at 180 min with no significant change at the end of digestion. Under the same conditions (duodenal digestion), TPC values for FHA increased to 180 min and then increased slightly at 240 min. Previous investigations showed that *in vitro* digestion decreased gallic and ferulic acid contents, in chokeberry juice, probably due to poor stability of these compounds to pH changes. However, flavonols, flavan-3-ols and caffeic acid derivatives were not affected by *in vitro* gastric digestion (Bermúdez-Soto et al., 2007[1]). While the stomach is not the major site for phenolic compounds absorption, small phenolic compounds are available or become available for absorption in the stomach. The duodenum and jejunum are the major sites of phenolic compounds absorption and transformation (Velderrain et al., 2014[38]). The tested extracts were transported from the stomach to the duodenum, and their pH values changes from acidic to alkaline due to neutralization of acid with bile and pancreatic juice. Depending on their chemical structure, phenolic compounds are unstable in alkaline conditions (Velderrain et al., 2014[38]). Bermúdez-Soto et al. (2007[1]) found in an *in vitro* duodenum digestion that flavonols, anthocyanins and flavan-3-ols, were unstable. This potential loss of phenolic compounds in the duodenal conditions accounts partially for the low bioavailability of some phenolic compounds, especially in FHR extract. The results of Tagliazucchi et al. (2010[35]), for the relative antioxidant activity of pure phenolic compounds individually subjected to simulated gastrointestinal digestion monitored by ABTS assay, showed that activity slightly decrease after gastric digestion. Pancreatic digestion causes increasing of antioxidant activity of gallic acid and catechin. This is maybe a reason for increasing of FHA relative activity in the third hour of incubation time. The results showed that the antioxidant activity of FHA and FHR was most likely stable in *in vitro* digestion system. The obtained results ensure that investigated extracts of *F. hexapetala*, which exhibited antioxidant activities *in vitro*, may display *in vivo* biological effects.

### Antimicrobial activity

The results of *in vitro* antibacterial activity of the extracts are given in Table 3(Tab. 3). The methanol extracts of *F. hexapetala*, both aerial parts and roots, revealed the highest antibacterial potential against *Escherichia coli* (ATCC 25922), where MIC values for FHA and FHR were 0.3125 and 0.625 mg/ml, respectively. Also, high antibacterial potential was observed against *Enterococcus faecalis* (MIC 1.25 and 2.5 mg/ml). The most resistant of bacteria species to the effect of selected extracts was *Pseudomonas aeruginosa* (ATCC 10145).

The results of antifungal activity are presented in Table 4(Tab. 4). *P. fastigiata* was the most sensitive fungal species tested against the extracts, showing MICs of 2.5 and 1.25 mg/ml for FHA and FHR, respectively, while *A. niger*, *A. alternata* and *D. stemonitis* were the most resistant species with MICs values above 10 mg/ml. Also, gallic acid and quercetin showed the highest antifungal activity against *P. fastigiata* (MIC 0.0156 and 0.5 mg/ml, respectively). The results indicate considerably lower antibacterial and antifungal potential of tested extract compared to the commercial antibiotic and antimycotics. However, FHA and FHR showed slightly lower antibacterial and antifungal potential compared to the gallic acid and quercetin activities. It can be noticed from the results for antibacterial and antifungal activity that the extract from aerial parts of* F. hexapetala*, compared to the root extract, demonstrated lower MIC values for all tested bacterial and fungal species, with exception of *A. niger*, *A. alternata* and *D. stemonitis*. As already been reported, the phenolic compounds play a key role in antimicrobial activity of plants (Silva and Fernandes, 2010[32]), so we can presume that FHA possess better MICs because of higher total phenolic content (Table 1(Tab. 1)). Also, FHA was richer in flavonoids and flavonols which have been shown to possess high antimicrobial activity (Cushnie and Lamb, 2005[3]).

The presented results of the antimicrobial assays for *F. hexapetala* extracts were performed and published for the first time. Several bacteria and fungi species used in antimicrobial assays in the current study reside in the group of food-borne pathogens, which posing a serious risk to the food safeness. Most of microbial pathogens that have infiltrated the food chain could provoke some of food-borne ailments, or their formation may be due to intoxication with bacteria or fungi toxins. Increasing usage of substantial assortment of wholesale antibiotics, to regulate infections and sicknesses in humans, may cause severe hypersensitivity backlash and lead to antibiotic resistance of human pathogens. Therefore, the scientific research aimed to find new antimicrobial compounds from medicinal plants are of great importance (Tajkarimi et al., 2010[36]). As a result of high specific activity of examined extracts, they could be exploited as an component of food products, as more natural substitute for the maintenance or postponement of product shelf life. By influencing on the food safety, they could prevent infections and diseases in humans.

## Conclusion

To our knowledge, this is the first detail report about antioxidant and antimicrobial activity of this important traditional medicinal plant. The results of presented study showed that methanolic extracts of aerial parts and roots of *F. hexapetala* possess remarkable antioxidant activities which could be related with high content of phenolic compounds (flavonoids in aerial parts of the plant and tannins in roots). Our results imply that above ground plant parts of *F. hexapetala* could be recommended for the human usage as a rich source of natural antioxidants. The extracts could act as antioxidants in different systems and efficiently inhibit lipid peroxidation in oil-in-water emulsion and when incorporated in the fresh meat system. In addition, their antioxidant activity remained almost unchanged or increased in a wide range of pH (1–9) after incubation for 1 h, during heating (100 °C) for 240 min and after gastrointestinal digestion. In words of food safety and food preservation, our findings suggest that *F. hexapetala* extracts may be effective as an antioxidant when present in foods subjected to baking, cooking, frying, microwaving, or high pH. Furthermore, as additives in food, these species could possible prevent or control growth of microorganisms, and thus improve the food preservation. Therefore, based on the exhibited results, the investigated extracts could serve as a potential source of natural antioxidants to be used as a protection from ROS affects, to prevent lipid peroxidation in food processing, to serve as the antimicrobial agents and to improve the biological properties of functional foods.

## Acknowledgements

This research was supported by the Ministry of Education and Science of the Republic of Serbia (project No. III 43004).

## Figures and Tables

**Table 1:**
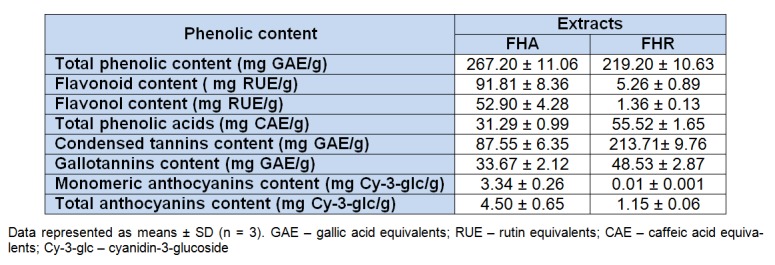
The phenolic contents of the methanolic extracts of *F. hexapetala* aerial parts (FHA) and roots (FHR)

**Table 2:**
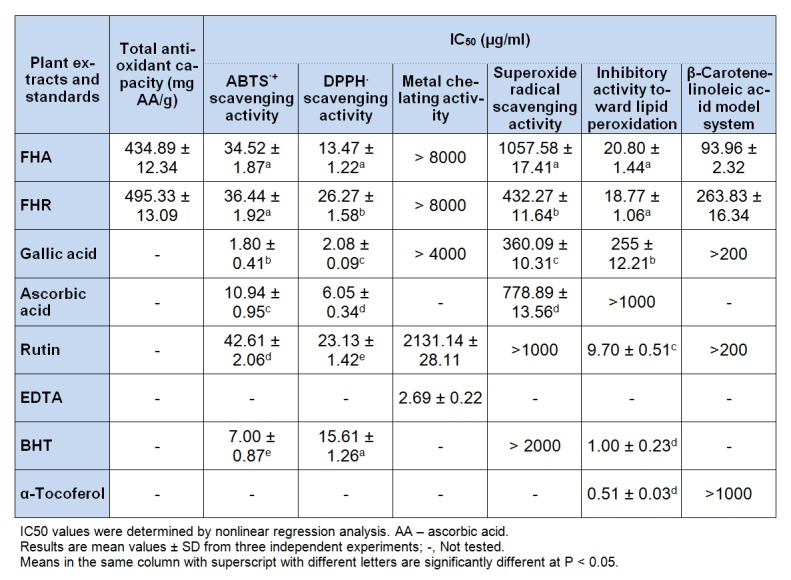
Total antioxidant capacity and IC_50_ values of antioxidant activities of the methanolic extracts from aerial parts (FHA) and roots (FHR) of *F. hexapetala*

**Table 3:**
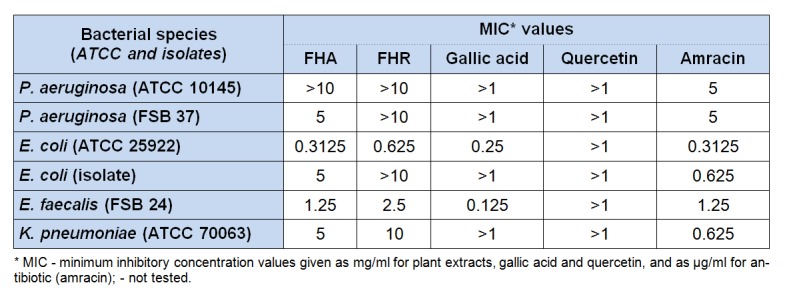
Antibacterial activity of *F. hexapetala* extracts (aerial parts and roots)

**Table 4:**
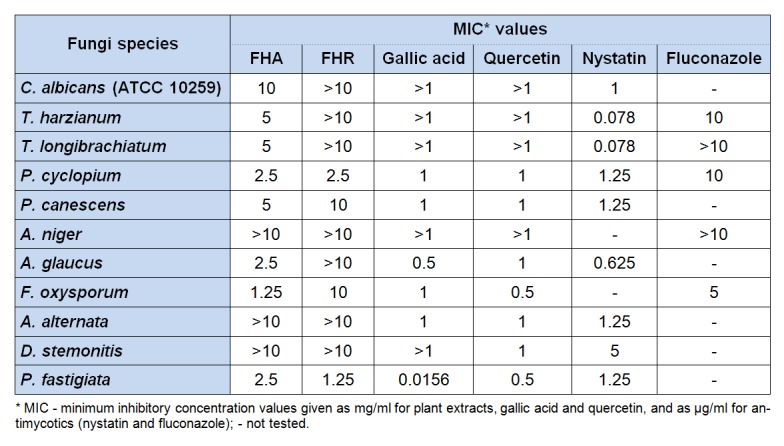
Antifungal activity of extracts from *F. hexapetala* compared to standard antimycotics
